# Understanding the role of small-molecule therapeutics for snake envenoming

**DOI:** 10.1016/j.ebiom.2025.105635

**Published:** 2025-03-06

**Authors:** Anjana Silva, Geoffrey Isbister

**Affiliations:** a Department of Parasitology, Faculty of Medicine and Allied Sciences, Rajarata University of Sri Lanka, Saliyapura, 50008, Sri Lanka; b Clinical Toxicology Research Group, University of Newcastle, Australia

We live in a world of disparity, where personalised medicine advances can solve complex conditions, while more basic health problems still pose considerable challenges to lower-income countries. Snake envenoming is a significant public health concern in the rural tropics and is closely associated with poverty, underdevelopment, and politically and socioeconomically marginalised communities. An accurate global estimate of snakebite burden is unavailable, but literature-based high estimates suggest approximately 5 million snakebites, 1.8 million snake envenomings and 90,000 deaths due to snakebite occur each year.^1^ Snake envenoming can cause life-threatening effects, including coagulopathy, neuromuscular paralysis, acute kidney injury and local tissue necrosis. In addition to acute effects, some snakebite survivors may live the rest of their lives with debilitating long-term effects that significantly lower their quality of life.^2^

Snake venoms are complex mixtures of enzymatic and non-enzymatic toxins that can cause diverse pathophysiological effects in snakebite patients. Only toxins from four primary protein families, phospholipases A_2_, snake venom metalloproteinases, snake venom serine proteases, and three-finger toxins, appear to cause most of the clinically important envenoming effects in humans.^3^ Many of these toxic effects are not readily reversible once they occur, including venom-induced consumption coagulopathy, presynaptic neuromuscular paralysis and local necrosis, which means that interventions need to be aimed at preventing or attenuating toxin-mediated effects. Importantly, there is a delay post-bite in which toxins reach the circulation, distribute to the target tissues and cause toxin-mediated tissue/organ injury prior to the appearance of clinical features. This is often described as a ‘pharmacokinetic-pharmacodynamic mismatch’.^4^^,^^5^ This means that specific treatment for snakebite should be initiated before there are clinically apparent effects of tissue or organ injury.

Antivenoms are the only specific treatment currently available and approved for snakebite. They are animal-derived antibodies or antibody fragments raised against snake venoms. They have been the standard of care for over 100 years despite very few clinical trials demonstrating effectiveness.^5^ A major problem with antivenoms is the high rate of early non-IgE mediated hypersensitivity reactions that occur, with anaphylaxis reported in over 40% of patients receiving antivenom in some.^6^ This risk and severity of antivenom reactions means that antivenoms need to be given in hospital by trained health care staff. In the rural tropics, where snakebites are common, bites occur in remote communities far from healthcare. Both the distance from healthcare and concerns about giving antivenom to patients without envenoming means there are significant delays in antivenom administration, limiting the prevention of irreversible toxin-mediated injury.^4^

Issues with antivenom safety, and additionally poor availability of antivenoms in many parts of the world, have meant that other treatments have been sought, with the aim to have cheaper and safer alternatives. Small molecule therapeutics are one such option that has emerged recently. Small molecules target a specific toxin protein family, such as phospholipase A_2_ toxins or snake venom metalloproteinases. They have a number of advantages, most importantly that some of them are repurposed treatments, which have undergone previous early trials in humans and appear to have few adverse effects. In addition, they are expected to have improved tissue penetration, minimal off-target toxicity, a broad therapeutic index, and the ability to be delivered orally. These characteristics make them suitable for use in more remote and resource-limited healthcare settings to prevent or minimise life-threatening effects before a snakebite victim reaches a hospital. One major drawback is that each small molecule will only inhibit one toxin type, so in snake species causing multiple toxin effects, multiple treatments are required.

Varespladib is a phospholipase A_2_ inhibitor that has shown pre-clinical efficacy against snake venom phospholipase A_2_s and is currently undergoing clinical trials.^7^ Marimastat is a broad-spectrum matrix metalloproteinase inhibitor that was initially developed as a cancer therapeutic and has also shown pre-clinical efficacy for local effects and coagulopathy induced by snake venoms.^8^ Unfortunately, neither of these small molecule therapeutics has received regulatory approval for their originally intended purpose, and both lack robust safety data. In contrast, unithiol is a temperature-stable and relatively inexpensive chelating agent that is routinely used to treat mercury, lead and arsenic poisoning, and, unlike antivenoms, is available in both oral and intravenous formulations. The oral formulation offers advantages for administration in the field, where the sterile equipment and trained medical personnel required for intravenous administration may be difficult to find. Unithiol has been shown to have pre-clinical efficacy against snake venom metalloproteinase activity, procoagulant activity and murine lethality for several carpet viper (genus *Echis*) venoms.^9^ In the March 2025 issue of *eBioMedicine*, Abouyannis and colleagues conducted a phase I trial on healthy Kenyan adults, characterising the pharmacokinetics of oral and intravenous unithiol, and demonstrating that a 1500 mg oral loading dose results in peak plasma concentrations similar to a 3 mg/kg intravenous dose.^10^ Based on the safety and pharmacokinetic data, the 1500 mg oral loading dose of unithiol, followed by 900 mg doses at 6 and 24 h has been recommended by the authors for the phase II clinical trials. This will hopefully provide the basis for further studies on human envenoming from specific snake species or genera and eventual approval for its routine use thereafter if effective.

While small-molecule therapeutics appear promising, their role as an initial and adjunct treatment in the management of snake envenoming will be determined by their clinical effectiveness for different medically important snakes and safety when used in snakebite patients. This will require well-designed and conducted clinical trials, with venom-specific and clinically meaningful outcomes of potential small molecule therapeutics that have been identified to have pre-clinical efficacy for particular snake species. Abouyannis and colleagues should be congratulated for conducting one of the first phase I studies of snakebite treatments, with plans for a phase II study in the future. Hopefully, studies like this will mean that small-molecule therapeutics will only be used in patients based on good-quality evidence, and will ultimately contribute to the World Health Organization's goal of halving global snakebite mortality and morbidity by 2030.

## Contributors

Anjana Silva: writing the original draft. Geoffrey Isbister: review and editing. Both authors read and approved the final version.

## Declaration of interests

Geoffrey Isbister received payment from Emergency Trauma Management Pty Ltd for consultation, writing and presenting of educational toxicology-specific material. Anjana Silva has no competing interests to declare.
